# Effects of Osimertinib Combined With Pulmonary Rehabilitation and Health Care Training on Pulmonary Function, Complications, and Quality of Life in Patients After Radical Resection of Lung Cancer

**DOI:** 10.3389/fpubh.2022.911377

**Published:** 2022-06-10

**Authors:** Haijiang Xu, Ruixia Guo, Yantao Yang

**Affiliations:** ^1^Department of Pharmacy, The First Affiliated Hospital of Zhengzhou University, Zhengzhou, China; ^2^Department of Gynecology, The First Affiliated Hospital of Zhengzhou University, Zhengzhou, China

**Keywords:** osimertinib, pulmonary rehabilitation and health care training, radical resection of lung cancer, pulmonary function, quality of life

## Abstract

**Objective:**

To explore the effects of osimertinib combined with pulmonary rehabilitation and health care training on pulmonary function, complications, and the quality of life (QOL) in patients after radical resection of lung cancer.

**Methods:**

The data of 120 patients with radical resection of lung cancer admitted to *The First Affiliated Hospital of Zhengzhou University* from February 2020 to February 2021 were retrospectively analyzed. According to the order of admission, they were equally divided into group p and group q. All patients were given pulmonary rehabilitation and health care training, and group p was treated with osimertinib, while group q received the treatment of pemetrexed combined with cisplatin. The pulmonary function, the incidence of complications, and QOL between the two groups were compared.

**Results:**

Compared with group q, the pulmonary function was higher (*P* < 0.001), the incidence of complications was significantly lower (*P* < 0.05), and QOL scores were markedly higher in group p after treatment (*P* < 0.001).

**Conclusion:**

The combination of osimertinib and pulmonary rehabilitation and health care training can improve the pulmonary function of patients with non-small cell lung cancer (NSCLC) with radical resection of lung cancer, and reduce their postoperative morbidity, thereby improving their QOL, which is conducive to reducing the patient's and society's medical burden.

## Introduction

Lung cancer is a malignant tumor originating from the mucosa or gland of the trachea and bronchus. According to the differences in histopathological characteristics, lung cancer is generally divided into non-small cell lung cancer (NSCLC) and small cell lung cancer (SCLC) in the clinic. NSCLC accounts for approximately 85% of all patients with lung cancer, while SCLC accounts for only 15% (1, 2). In recent years, with changes in the social environment and living habits, the clinical characteristics of lung cancer have seen a substantial transformation in China (3, 4), but the main treatment option of patients has not been affected. Surgery is still the main treatment for patients with early, medium, and some advanced NSCLC, and 90% of patients are supplemented with chemotherapy after surgery (5) to improve QOL. At present, chemotherapy has an exact effect on SCLC, and about 1% of patients can be cured by chemotherapy (6). However, the effects of chemotherapy in patients with NSCLC are unsatisfactory, and patients can only relieve the clinical symptoms through chemotherapy drugs, which cannot effectively improve pulmonary function (7). For patients with NSCLC with the EGFR gene mutation, the T790M mutation will lead to an increase in chemoresistance, and patients who are insensitive to the conventional pemetrexed often get worse after treatment (8). In recent years, the third generation of epidermal growth factor receptor tyrosine kinase inhibitor (EGFR-TKI) developed by AstraZeneca has been used in the treatment of the NSCLC with the EGFR gene mutation. The drug has a significant therapeutic effect on the central nervous system with mild toxicity and favorable safety. The postoperative rehabilitation and health care training based on medication can effectively improve the endurance and strength of patients' respiratory muscles, thereby reducing the bodily oxygen consumption, enhancing the cardiopulmonary and muscular function, and providing a benign physiological basis for medication (9) and improving the patients' QOL. There is no study on the effects of osimertinib combined with pulmonary rehabilitation and health care training on patients with NSCLC with thee radical resection of lung cancer by reviewing the previous literature. Based on this, 120 patients were selected to explore the actual effects of osimertinib combined with pulmonary rehabilitation and health care training. The reports are as follows.

## Materials and Methods

### Research Design

This retrospective study was conducted under the guidance of the Declaration of Helsinki (2013) (10) to explore the effects of osimertinib combined with pulmonary rehabilitation and health care training on pulmonary function, complications, and QOL in patients after radical resection of lung cancer. The blind level of this study was double-blind. The study subjects and researchers did not know the grouping of this experiment, and the research designers were responsible for arranging and controlling the experiment. This study was approved by the ethics committee of The First Affiliated Hospital of Zhengzhou University.

### General Information

The data of patients with radical resection of lung cancer admitted to *The First Affiliated Hospital of Zhengzhou University* from February 2020 to February 2021 were retrospectively analyzed, and the patients were included according to the following criteria: (1) Patients with the pulmonary CT examination as punctiform or patchy shadows who were diagnosed with lung cancer by pathological examination, which met the diagnostic criteria in the Chinese guidelines on the diagnosis and treatment of primary lung cancer (2015 version) (11); (2) Patients who had the EGFR T790M mutation-positive non-small cell lung cancer (12); (3) Patients who had surgical indications for radical resection of lung cancer; (4) Patients who had no disease affecting the motor nerve, joint, and muscle; (5) Patients who were not malnourished; (6) Patients who had an expected survival time of more than 6 months; (7) The Karnofsky performance status score was more than 60 points in patients; (8) The age of the patients was more than 18 years old; and (9) Patients who had good tolerance and compliance. In the following circumstances, patients were not included in this study: (1) Patients with some factors such as hearing impairment, language disorders, unconsciousness or mental illness, and patients who could not communicate with others; (2) Patients with a grave organic disease or dysfunction in vital organs; (3) Patients with complications, such as pulmonary infection and respiratory failure; (4) Patients with malnutrition or excessive obesity; (5) Patients with the expected survival period <6 months; and (6) Patients with the training participation rate <80%.

All 120 patients included in this study were aware of the purpose, significance, content, and confidentiality of this study and they signed their informed consent. They were equally divided into group p and group q according to the order of admission. There was no significant difference in general information between the two groups (*P* > 0.05), which can be used as the research subjects. See details in Table 1.

**Table 1:** Comparison of patients' general information.

**Groups**	**Group p**	**Group q**	**X^2^/t**	***P***
	**(*n =* 60)**	**(*n =* 60)**		
Male/female	32/28	34/26	0.135	0.714
Age (years)	52.75 ± 8.67	53.35 ± 8.40	0.385	0.701
Body mass (kg)	64.98 ± 2.41	64.77 ± 2.56	0.463	0.645
BMI (kg/m^2^)	22.14 ± 1.65	22.10 ± 1.47	0.140	0.889
Pathological types			0.035	0.853
Squamous cell carcinoma	24	25		
Adenocarcinoma	36	35		
TNM staging			0.039	0.843
III	41	42		
IV	19	18		
Surgical procedures			0.035	0.853
Thoracic surgery	24	25		
Video-assisted thoracoscopic surgery	36	35		
Resection range				
Single lobectomy	25	24	0.035	0.853
Bilobectomy	15	16	0.044	0.835
Wedge resection	14	15	0.046	0.831
Sleeve resection	6	5	0.100	0.752
Educational level				
Primary school	12	14	0.196	0.658
Senior middle school	32	30	0.134	0.715
University	16	16	0.000	1.000
Monthly profit (yuan)			0.141	0.707
≥4000	22	24		
<4000	38	36		
Living habits				
Smoking history	40	38	0.147	0.702
Drinking history	32	30	0.134	0.715
Place of residence			0.034	0.854
Town	26	27		
Countryside	34	33		

### Standards of Withdrawal From Experiment

In the following conditions, the study group judged that the patients were unable to continue the experiment, and their case record forms were retained, but the data analysis was not performed. These conditions were: (1) Patients who had sudden exacerbation during the experiment and (2) In the experiment, subjects who were unwilling to continue the clinical trials, and who applied to the study group for withdrawal from the experiment.

### Methods

All patients were given pulmonary rehabilitation and health care training. A team on rehabilitation and health care training was constructed, and the team members included the attending physicians, supervisor nurses, and primary nurses. After admission, the primary nurses collected the clinical information of patients, carried out the health promotion for patients, and improved their training compliance. The team on rehabilitation and health care training developed programs of the pulmonary rehabilitation's exercise training based on the actual circumstances of the patients. The patients were given deep pursed lips breathing at 1–2 weeks after discharge, with a daily training for 0.5 h. The deep pursed lips breathing meant that the patients inhaled slowly with the nose until the gas could not be inhaled, and then exhaled slowly with the mouth in contracted lips after 5 s. The relaxation and contraction exercise in muscles of the whole body was implemented in the third week after discharge, with daily training for 0.5 h. The 1–3 weeks of training programs were repeated in the fourth week, with daily training for 1 h. On Wednesday and Sunday, the centralized training was performed. At first, the patients were asked to repeat the deep pursed lips breathing, followed by progressive respiratory muscle contractions. After the patients took a semi-recumbent position, they took a deep breath slowly for three times and then held their breath, and they should contract the respiratory and abdominal muscles. When the patients could not persist, they could exhale quickly, and then gradually relax the muscle groups, and finally contract the muscles of the whole body. After the patients took a semi-recumbent position, the muscle contraction was performed from far to near and from top to bottom. Patients stretched themselves for 5 s with the taut muscles, and the muscles of the whole body were relaxed. In addition, the patients could jog according to the actual situation, first walking slowly at a speed of 30 steps/min, then walking fast at a speed of 75 steps/min, and finally jogging.

In addition, group p was treated with the osimertinib, and they were given 80 mg of osimertinib mesylate (AstraZeneca Pharmaceutical Co., Ltd.; specification: 80 mg; NMPA approval No.: J20180027) once a day. Group q received the treatment of pemetrexed and cisplatin. The 500 mg/m^2^ of pemetrexed disodium (Sichuan Huiyu Pharmaceutical Co., Ltd.; specification: 500mg; NMPA approval No.: H20173301) was intravenously infused on the first day, and 75 mg/m^2^ of cisplatin (Shandong Luoxin Pharmaceutical Group Co., Ltd.; specification: 10 mg; NMPA approval No.: H20046375) was intravenously infused on the second day with 3 weeks as 1 cycle. The routine examinations, such as blood routine, hepatorenal function, electrocardiogram, and dexamethasone (Guangdong Luofushan China National Medicines Co., Ltd.; specification: 0.75 mg; NMPA approval No.: H44024841) and folic acid tablets (Jiangxi Pharmaceutical Co., Ltd.; 0.4 mg; NMPA approval No.: H36020872) were given before chemotherapy.

### Observational Criteria

#### General Information

The patients' data on social demography and clinic were collected including gender, age, body mass, BMI, pathological types, TNM staging (13), surgical procedures, resection range, educational level, monthly profit, living habits, and place of residence.

#### Pulmonary Function

The forced expiratory volume in one second (FEV_1_), forced vital capacity (FVC), FEV_1_/FVC, the percentage of maximal ventilatory volume in predicted value (MVV%), and maximal vital capacity were recorded before treatment and at 1 month after treatment using a pulmonary function instrument [Body Plethysmograph, NMPA (I) 20172071024] in Medical Graphics Corporation.

#### Incidence of Complications

The types of complications were recorded, the number of patients with complications was counted, and the incidence of complications was calculated. The incidence of complications = (the number of patients with complications/total cases) ×100%.

#### QOL

The QOL instruments for patients with cancer: lung cancer (QLICP-LU) (14) developed by Wan Chonghua et al. was used to investigate patients' QOL before treatment and 1 month after treatment. The scale included the somatic function (7–35 points), psychological function (12–60 points), social function (6–30 points), common symptoms and adverse reactions (7–35 points), and specific modules (8–40 points) with the test-retest reliability as 0.78. The α value of internal reliability in various fields and the test-retest reliability were above 0.70, which had favorable reliability and validity.

### Statistical Treatment

In this study, the experimental data were processed by SPSS20.0, and GraphPad Prism 7 (GraphPad Software, San Diego, USA) was used to draw pictures of the data. The items included in the study were enumeration data and measurement data tested by x^2^ and t–tests. When *p* < 0.05, the differences were considered to be statistically significant.

## Results

### Comparison of Patients' General Information

There was no statistical difference in the general information between the two groups (*P* > 0.05). See details in Table 1.

### Comparison of Patients' Pulmonary Function

**Figure 1:**
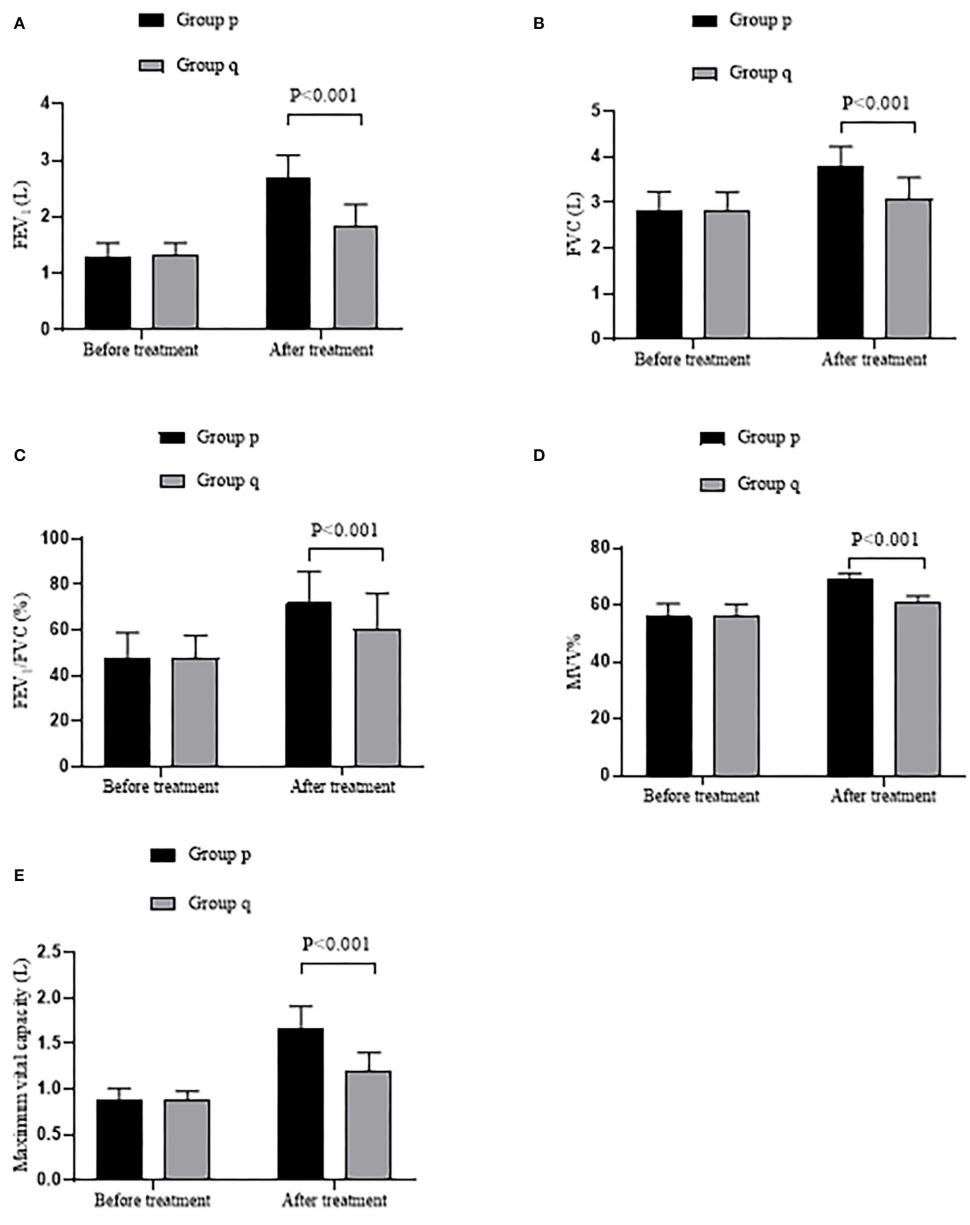
Comparison of patients' pulmonary function (x ± s). **(A)** showed the FEV_1_ (L). **(B)** showed the FVC (L). **(C)** showed the FEV_1_/FVC (%). **(D)** showed the MVV%. **(E)** showed the maximal vital capacity (L).

Compared with group q, the pulmonary function was observably higher in group p after treatment (*P* < 0.001). See details in Figure 1.

Compared with group q, the FEV_1_ level, FVC level, FEV_1_/FVC level, MVV% level, and maximal vital capacity were observably higher in group p after treatment (2.68 ± 0.41 vs. 1.82 ± 0.40, 3.78 ± 0.45 vs. 3.08 ± 0.47, 71.82 ± 13.98 vs. 60.41 ± 15.75, 68.98 ± 2.10 vs. 60.77 ± 2.47, and 1.67 ± 0.24 vs. 1.21 ± 0.19, all *P* < 0.001), with no significant difference before treatment (1.30 ± 0.24 vs. 1.32 ± 0.22, 2.80 ± 0.44 vs. 2.81 ± 0.42, 47.73 ± 11.17 vs. 47.74 ± 9.96, 55.98 ± 4.50 vs. 55.90 ± 4.48, and 0.89 ± 0.12 vs.0.88 ± 0.10, all *P* > 0.05).

### Comparison of Patients' Incidence of Complications

Compared with group q, the incidence of complications in group p was significantly lower (*P* < 0.05). See details in Table 2.

**Table 2:** Comparison of patients' incidence of complications [n (%)].

**Groups**	**Group p**	**Group q**	**X^2^**	***P***
	**(*n =* 60)**	**(*n =* 60)**		
Pulmonary infection	0 (0.0)	1 (1.7)	1.008	0.315
Dyspnea	2 (3.3)	5 (8.3)	1.365	0.243
Atelectasis	2 (3.3)	4 (6.7)	0.702	0.402
Respiratory failure	0 (0.0)	1 (1.7)	1.008	0.315
Eating disorder	0 (0.0)	1 (1.7)	1.008	0.315
The number of occurrences	4 (6.7)	12 (20.0)	4.615	0.032

### Comparison of Patients' QOL

**Figure 2:**
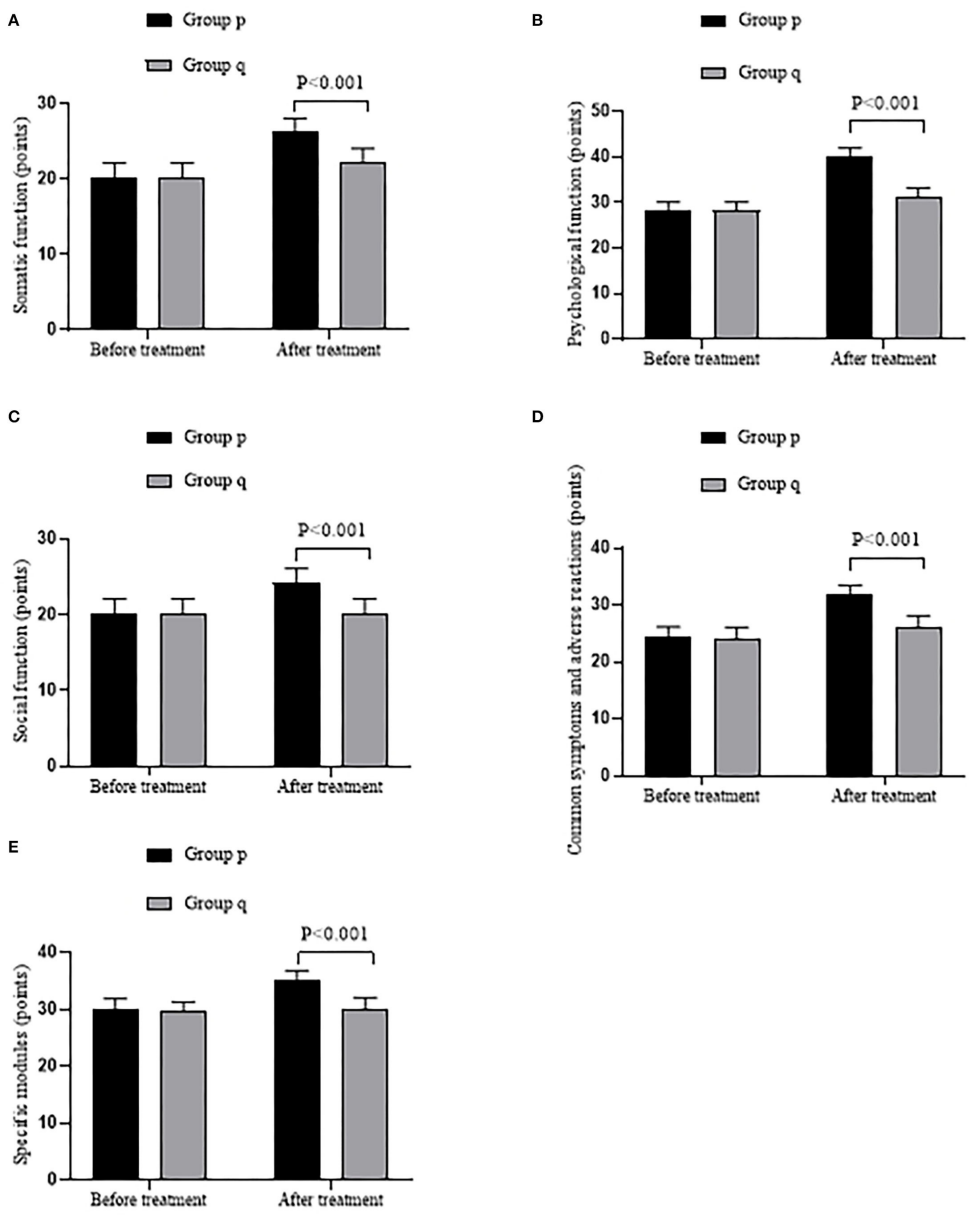
Comparison of patient's QOL ($\bar{x}$ ± s, points). **(A)** showed the somatic function. **(B)** showed the psychological function. **(C)** showed the social function. **(D)** showed the common symptoms and adverse reactions. **(E)** showed the specific modules.

Compared with group q, QOL scores in group p were observably higher after treatment (*P* < 0.001). See details in Figure 2.

Compared with group q, the somatic function score, psychological function score, social function score, the score of common symptoms and adverse reactions, and the score of specific modules were markedly higher in group p after treatment (26.20 ± 1.90 vs. 22.20 ± 1.91, 40.20 ± 1.84 vs. 31.27 ± 1.97, 24.17 ± 1.92 vs. 20.23 ± 1.89, 31.78 ± 1.66 vs. 26.22 ± 1.93, and 35.12 ± 1.70 vs. 30.18 ± 1.92, all *P* < 0.001), with no significant difference before treatment (20.17 ± 2 vs. 20.23 ± 1.95, 28.27 ± 1.85 vs. 28.25 ± 1.89, 20.18 ± 1.96 vs. 20.20 ± 1.95, 24.25 ± 1.93 vs. 24.18 ± 1.94, and 29.95 ± 1.93 vs. 29.58 ± 1.75, all *P* > 0.05).

## Discussion

According to the epidemiological data in 2018, the incidence and mortality of lung cancer ranked first in the malignant tumors in China. About 1.8 million patients died from lung cancer each year, accounting for 18.4% of all cancer death cases (15, 16). After the pathological types are clarified, the comprehensive treatment measures for patients can effectively reduce their mortality and prolong their survival period. The comprehensive treatment measures mainly include surgery, chemotherapy, and radiotherapy. Since SCLC often has metastasis in the early and middle stages, chemotherapy and radiotherapy were generally adopted in the clinic (17), while patients with NSCLC with a limited disease are often treated with surgery and postoperative radiotherapy and chemotherapy. Although radical resection of lung cancer can remove the lesions of lung cancer and reduce the clinical symptoms of patients with NSCLC, it cannot effectively improve the pulmonary function of patients (18, 19). In this study, patients were given pulmonary rehabilitation and health care training after the radical resection of lung cancer. The training methods mainly included subjective testing, breath instruction, and intensive training, which could regulate the respiratory contraction motion of patients to the maximum extent, improve the function of tracheal epithelium, and enhance the elimination effect of airway ciliary, thereby reducing the incidence of complications such as respiratory tract infection. In the process of the repeated exercises, patients could produce sufficient compensative capacity and enhance their lung capacity and lung function (19). Therefore, the pulmonary function indexes such as FEV_1_ and FVC in the two groups after treatment were improved. Scholars Van Haran Robert M et al. have found that the pulmonary function indexes, such as FEV_1_ and FVC of patients, were significantly improved by the application of the pulmonary rehabilitation and health care training in the rapid rehabilitation of lung cancer patients after surgery, indicating that the rehabilitation training method has a clear effect on lung cancer patients (20). However, the patients in group p had a more significant increase in pulmonary function compared with those in group q. The reason is that the pemetrexed used in group q has an anti-tumor effect, but the therapeutic effect on NSCLC is not obvious, while the osimertinib used in group p has a targeted therapeutic effect on the patients with NSCLC with EGFR gene mutation.

In general, postoperative chemotherapy for patients with SCLC can often obtain an ideal therapeutic effect, but the chemotherapy effect in patients with NSCLC is not very clear,; patients with the feeble constitution, poor bone marrow function, or dysfunction in vital organs are especially unsuitable for chemotherapy (21–24). Patients with NSCLC with the EGFR gene mutation even get worse after chemotherapy, so patients with NSCLC should be careful with chemotherapy and look for more effective treatment methods. Osimertinib, a third generation of EGFR-TKI, is the primary therapeutic drug after patients have drug resistance to the first and second generation of targeted drugs. Phase I and III clinical trials have shown that simertinib is effective in patients with locally advanced or metastatic EGFR mutation-positive non-small-cell lung cancer (25). After the previous EGFR-TKI treatment, patients in the disease progression received the simertinib, with a disease control rate of 92.0%. Therefore, osimertinib is certified as a ground-breaking therapy drug for NSCLC by the FDA (26, 27). The mechanism of osimertinib is that the acrylamide contained in osimertinib can form the covalent bonding with C797 at the edge of the ATP binding site in the catalytic domain of the EGFR gene, and then the irreversible binding occurs with the specific EGFR mutations, to solve the problem of abnormal signal transduction pathway in downstream cells caused by EGFR mutation, inhibit the excessive proliferation and mutation of tumor cells due to the EGFR mutation, and reduce the metastasis rate. The adverse reactions of osimertinib are mild compared with the conventional chemotherapeutic agents such as pemetrexed, and osimertinib shows a better tolerance in most clinical trials. Zhao Yang et al. have found that patients with osimertinib have no toxic reaction at a dose of 240 mg/d, and the incidence of severe adverse reactions is only 2%. Therefore, patients treated with osimertinib have a better organismal tolerance and an ideal long-term QOL (28). Chul et al. have confirmed that patients treated with osimertinib have significant progress in psychological and social functions, with a significant improvement in QOL (29), and the results are in accordance with this study. It is worth noting that the effect of osimertinib in improving the QOL of patients was mostly studied individually in current academia. This study paid attention to the combined application of osimertinib and other treatment measures, which found that osimertinib could improve the effect of pulmonary function training, and the higher QOL score in group p was consistent with the argument.

It is worth noting that long-term use of osimertinib will also produce drug resistance. No drug is available for patients with osimertinib resistance in the current practice. Scholars Xueting et al. have found that dihydroartemisinin can overcome the drug resistance (30), but there still lacks a large number of control studies. The fourth generation of EGFR-TKI drugs needs to be developed with osimertinib as a breakthrough point. At present, patients should be given the osimertinib combined with the pulmonary rehabilitation and health care training in the clinic, thereby providing a favorable physiological basis for the treatment of patients, reducing the postoperative morbidity, and improving their QOL.

In conclusion, the combination of osimertinib and pulmonary rehabilitation and health care training can improve the pulmonary function of patients with NSCLC with radical resection of lung cancer, and reduce the postoperative morbidity, thereby improving their QOL, which is conducive to reducing the patient's and society's medical burden.

## Data Availability Statement

The original contributions presented in the study are included in the article/supplementary material, further inquiries can be directed to the corresponding author.

## Ethics Statement

The studies involving human participants were reviewed and approved by The First Affiliated Hospital of Zhengzhou University. The patients/participants provided their written informed consent to participate in this study. Written informed consent was obtained from the individual(s) for the publication of any potentially identifiable images or data included in this article.

## Author Contributions

HX and YY: conception, design, data analysis, and interpretation. All authors: administrative support, provision of study materials or patients, collection and assembly of data, manuscript writing, and final approval of manuscript.

## Conflict of Interest

The authors declare that the research was conducted in the absence of any commercial or financial relationships that could be construed as a potential conflict of interest.

## Publisher's Note

All claims expressed in this article are solely those of the authors and do not necessarily represent those of their affiliated organizations, or those of the publisher, the editors and the reviewers. Any product that may be evaluated in this article, or claim that may be made by its manufacturer, is not guaranteed or endorsed by the publisher.
